# Comparison of ultrasound-guided internal jugular vein and supraclavicular subclavian vein catheterization in critically ill patients: a prospective, randomized clinical trial

**DOI:** 10.1186/s13613-022-01065-x

**Published:** 2022-10-01

**Authors:** Becem Trabelsi, Zied Hajjej, Dhouha Drira, Azza Yedes, Iheb Labbene, Mustapha Ferjani, Mechaal Ben Ali

**Affiliations:** 1 Department of Anesthesiology and Critical Care Medicine, Faculty of Medicine of Tunis, Taher Maamouri Teaching Hospital of Nabeul, University of Tunis El Manar, 8000 Nabeul, Tunisia; 2 Department of Anesthesiology and Critical Care Medicine, Faculty of Medicine of Tunis, Military Hospital of Tunis, University of Tunis El Manar, 1008 Montfleury, LR12DN01 Tunis, Tunisia

**Keywords:** Internal jugular vein, Subclavian vein, Supraclavicular approach, Ultrasound guidance, Scanning axis, Central venous cannulation

## Abstract

**Background:**

The aim of this study was to compare the effectiveness and safety of ultrasound-guided out-of-plane internal jugular vein (OOP-IJV) and in-plane supraclavicular subclavian vein (IP-SSCV) catheterization in adult intensive care unit.

**Methods:**

A total of 250 consecutive patients requiring central venous catheterization, were randomly assigned to undergo either ultrasound-guided OOP-IJV or IP-SSCV cannulation. All catheterizations were carried out by three physicians. The primary outcome was the first attempt success rate. Ultrasound scanning time, venous puncture time, insertion time, overall access time, number of puncture attempts, number of needle redirections, success rate, guidewire advancing difficulties, venous collapse and adverse events were also documented.

**Results:**

The first attempt success rate was significantly higher in IP-SSCV group (83.2%) compared to OOP-IJV group (63.2%) (*p* = 0.001). The IP-SSCV group was associated with a longer ultrasound scanning time (16.54 ± 13.51 vs. 5.26 ± 4.05 s; *p* < 0.001) and a shorter insertion time (43.98 ± 26.77 vs. 53.12 ± 40.21 s; *p* = 0.038). In the IP-SCCV group, we recorded a fewer number of puncture attempts (1.16 ± 0.39 vs. 1.47 ± 0.71; *p* < 0.001), needle redirections (0.69 ± 0.58 vs. 1.17 ± 0.95; *p* < 0.001), difficulties in guidewire advancement (2.4% vs. 27.4%; *p* < 0.001), venous collapse (2.4%, vs. 18.4%; *p* < 0.001) and adverse events (8.8% vs. 13.6%; *p* = 0.22).

**Conclusions:**

The IP-SSCV approach is an effective and a safe alternative to the classic OOP-IJV catheterization in critical adult patients.

*Trial registration*: Clinicaltrials.gov, NCT03879954. Registered March 19, 2019—Retrospectively registered, https://clinicaltrials.gov/ct2/show/NCT03879954.

**Supplementary Information:**

The online version contains supplementary material available at 10.1186/s13613-022-01065-x.

## Background

The central venous catheter (CVC) placement is one of the most commonly performed invasive procedures for the management of critically ill patients [1] with two main venous routes namely the internal jugular vein (IJV) and subclavian vein (SCV). The CVC placement can lead to potentially serious adverse events [2]. The use of bedside ultrasound (US) guidance has been shown to facilitate the CVC insertion and to reduce the number of procedural-related complications [3, 4]. The role of US-guidance for IJV cannulation is currently well established [5] and most studies have used the short-axis view in combination with out-of-plane (OOP) needle approach [6].

The US-guided SCV cannulation via the infraclavicular (IC) approach is technically more challenging because of the acoustic shadow of the overlying clavicle [7]. Therefore, insufficient evidence has been found to recommend the use of US-guidance for SCV cannulation [5, 8, 9] despite of many advantages of SCV over IJV route including its larger diameter, ability to remain patent even in situations of severe hypovolemia, lower risk of central line-associated blood stream infection and thrombosis, increased patient comfort and accessibility in case of cervical spine trauma [10, 11]. The supraclavicular approach, an underused technique, was first described by Yoffa in 1965 as an alternative to the IC approach for SCV cannulation [12]. It offers a better sonographic visualization of the SCV using the long-axis imaging technique and allows an in-plane (IP) needle approach [10].

To the best of our knowledge, there are no published studies comparing the short-axis OOP-IJV and the long-axis IP supraclavicular SCV (IP-SSCV) cannulation in adults. Hence, the purpose of this study was to compare the effectiveness and safety of OOP-IJV and IP-SSCV approaches for US-guided CVC placement in adult intensive care unit (ICU).

## Patients and methods

### Study design and participants

This prospective randomized clinical trial was conducted in a 12-bed medical-surgical ICU at the Teaching Hospital of Nabeul (Tunisia) between February 2019 and November 2019. It was approved by the local ethics committee and was retrospectively registered in the ClinicalTrials.gov database (NCT03879954). Patients older than 18 years requiring first CVC insertion were enrolled after obtaining a written informed consent from the patient or the patient’s closest relative. They were randomly divided according to a computer generated randomization table (Random Allocation Software 2.0) into two groups: US-guided OOP-IJV and IP-SSCV catheterization (1:1 ratio). Randomization was created with simple allocation to the two study groups based on the opaque, sealed envelope technique. A physician not involved in the study performed the randomization. The exclusion criteria were major blood coagulation disorders, any thrombotic formations within the vein, congenital or acquired deformity of neck or clavicle and cannulation site infection, hematoma or surgery.

### Methods

All catheterizations were carried out by three anesthesiology residents. Each of whom had three years of experience in anesthesia and intensive care. Standard monitoring devices including electrocardiography, pulse oximetry and non-invasive blood pressure were applied. When patients did not have a pre-existing CVC, a peripheral venous access was obtained. Patients were placed in 10° Trendelenburg position to avoid air embolism and to distend the vein. The head was slightly turned toward the opposite side of venipuncture and the arm was kept to the side.

The operator stood at the head of the patient for IJV cannulation and beside the patient for SCV cannulation using supraclavicular approach. The US screen was placed in the operator’s line of sight during needle insertion [3]. Complete aseptic technique was employed. Local infiltration was made at the puncture site with 5 ml of 1% lidocaine in conscious patients.

### US technique

The investigators used a 7Fr triple lumen CVC (Certofix^®^ Trio S720, B. Braun, Melsungen AG, Germany) and an US unit (Esaote MyLab™ X5, Genova, Italy) with high-frequency linear array transducer (15 MHz) inserted in a sterile probe cover containing an US gel.

**Fig. 1:**
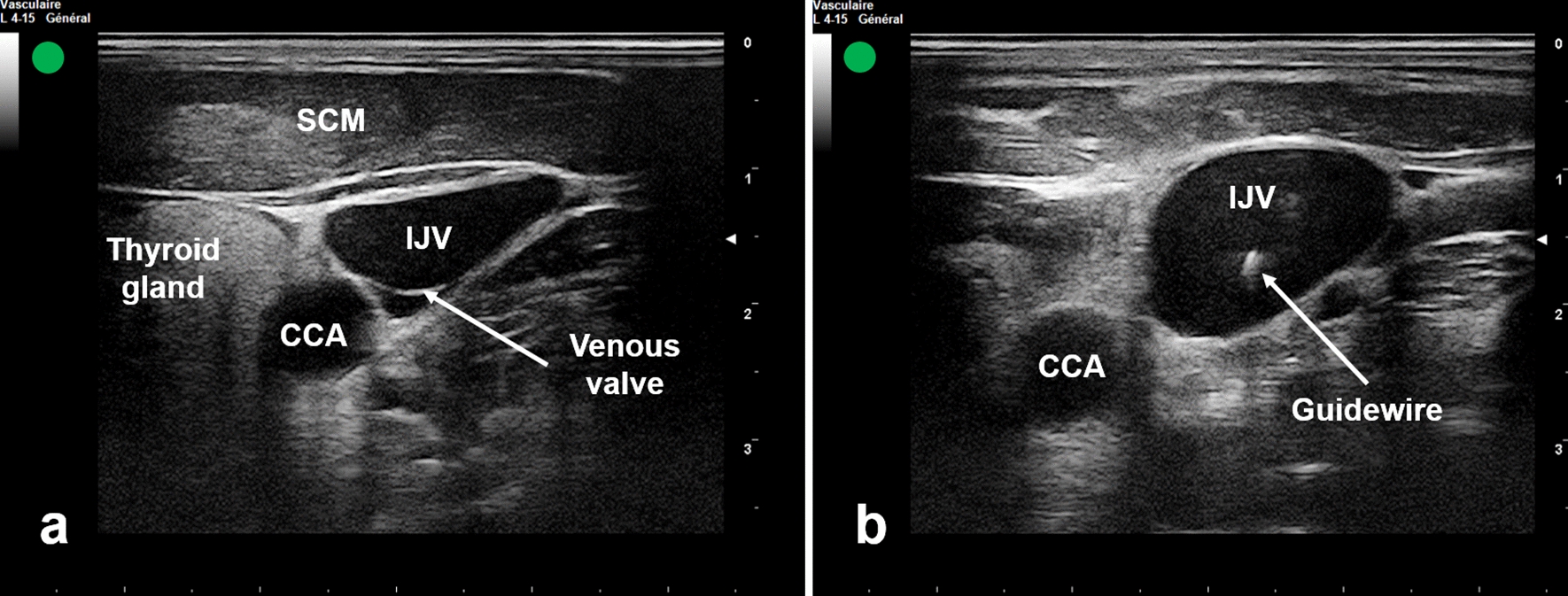
Ultrasound-guided IJV catheterization using the short-axis view of the vein in combination with out-of-plane needle approach. **a** Ultrasound visualization of IJV and CCA.** b** Ultrasound visualization of the guidewire in the IJV. *IJV* internal jugular vein, *CCA* common carotid artery, *SCM* sternocleidomastoid muscle

For OOP-IJV cannulation, the transducer was placed on transverse position over the patient’s neck at the level of cricoid cartilage to identify IJV and common carotid artery (CCA) in short-axis view (Fig. 1a). The CCA and the IJV were differentiated by pulsatility of the artery, compressibility of the vein and if necessary through pulsed Doppler control of vascular flux. The vein was then centered on the screen. The skin puncture was made in the center of the US image using a needle attached to a syringe. The needle was introduced at an angle of 60° to the skin surface, perpendicular to the transducer. Afterwards, the needle was advanced toward IJV while monitoring tissue deformation under US-guidance. When the operator noticed an indentation of the anterior wall of the vein, additional pressure was applied until disappearance of the vein deformation and visualizing an echogenic point in the center of the vein.

**Fig. 2:**
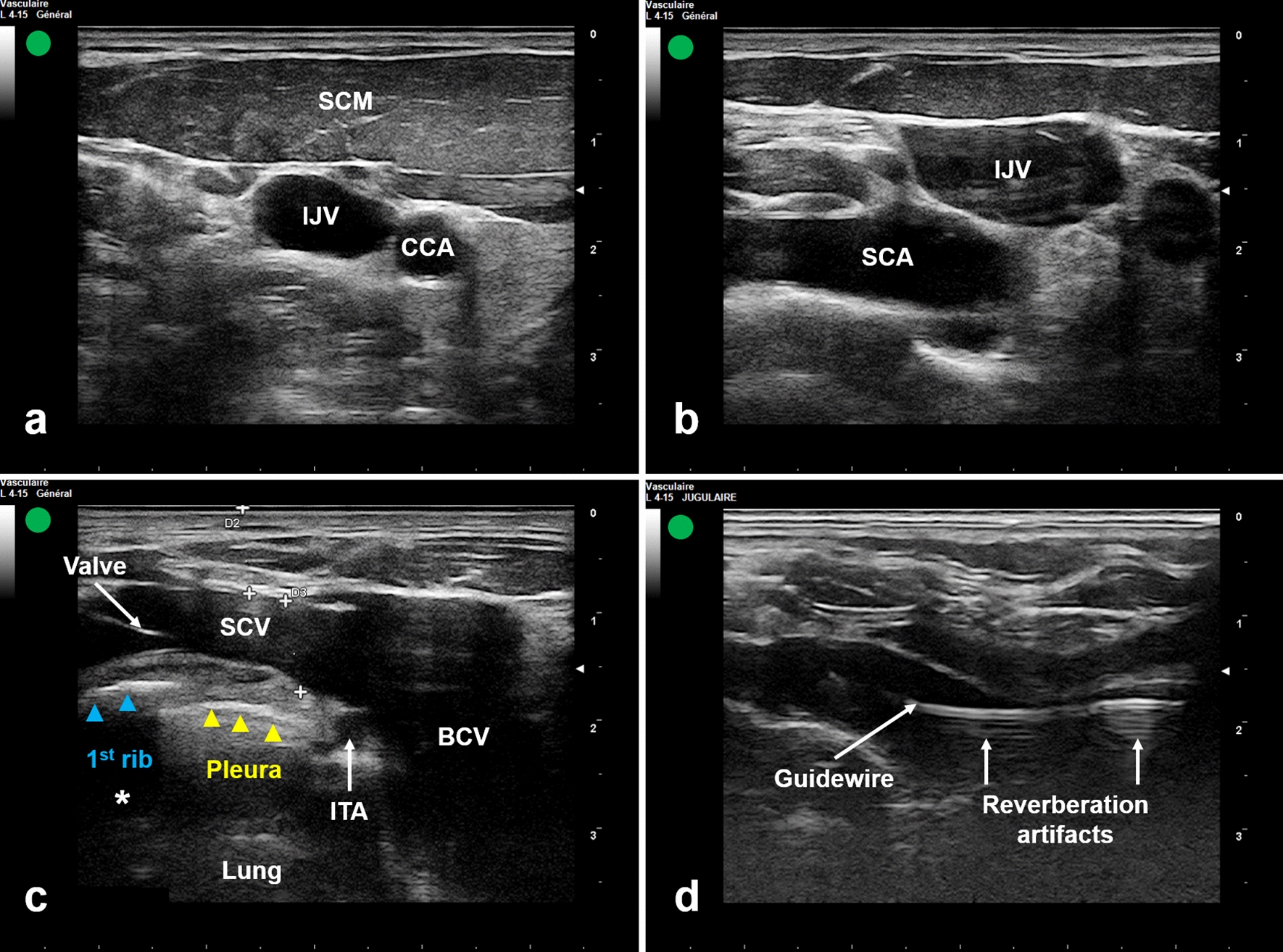
Ultrasound-guided subclavian vein catheterization using the long-axis view of the vein via the supraclavicular approach in combination with in-plane needle approach. **a** Ultrasound short-axis view of IJV. **b** Ultrasound identification of IJV and SCA in the supraclavicular fossa. **c** Ultrasound long-axis view of SCV and BCV. **d** Ultrasound visualization of the guidewire in the SCV. *SCV* subclavian vein, *SCA* subclavian artery, *BCV* brachiocephalic vein, *ITA* internal thoracic artery, *Asterisk* acoustic shadow of the 1st rib

For IP-SSCV cannulation, a short-axis view of IJV was obtained first (Fig. 2a). The probe was slid caudally following IJV until the junction of SCV and IJV was reached in the supraclavicular fossa. At this level, the subclavian artery should be identified in order to avoid arterial puncture (Fig. 2b). The probe was then tilted anteriorly and turned slightly to get the best long-axis view of the SCV and the brachiocephalic vein (BCV). The latter is formed by the confluence of SCV and IJV. The SCV was confirmed by its location anterior to the artery and its direct contact with the underlying pleura [13] (Fig. 2c). The nature of the vein could also be confirmed by pulsed Doppler. According to an IP approach, the needle attached to a syringe was inserted at the base of the transducer at a 30° angle. The needle was advanced from lateral to medial. The needle tip was then guided under real-time US-guidance targeting SCV (Additional file 1).

In both groups, catheterization was done through Seldinger technique. After successful blood aspiration, a J-shaped guidewire was smoothly advanced through the needle into the vein. Subsequent to needle removal, US was used to confirm the correct location of the guidewire into the target vein (Figs. 1b, 2d). The venous cannulation was completed as usual and all ports of the CVC were checked for free flow of blood. If the guidewire could not be advanced due to resistance, the needle and the guidewire were withdrawn and subsequent re-puncture should be considered as described previously. The right side was the preferred catheter location because of the absence of the thoracic duct [14] and the less risk of pneumothorax since the pleural dome is lower on this side [9]. In addition, the right IJV has a straighter pathway to the superior vena cava [15]. A post-procedure chest X-ray was taken to confirm the placement of the catheter and to check for any complications.

### Primary and secondary outcomes

The primary outcome was the first attempt success rate defined as the proportion of the correct placement of the guidewire into the intended vein with single skin puncture.

The secondary outcomes were: (1) the US scanning time (defined as the time required for US scanning of the vein); (2) the venous puncture time (recorded from the first skin puncture to venous blood aspiration); (3) the insertion time (recorded from the first skin puncture to the US confirmation of the correct position of the guidewire into the target vein); (4) the overall access time (defined as the time between the beginning of the US scanning and the US confirmation of the correct position of the guidewire; the time following the US verification of the guidewire position was not considered because it does not depend on the US technique); (5) the number of puncture attempts (defined as the average number of separate skin punctures); (6) the number of needle redirections; (7) the success rate (defined as the proportion of the correct placement of the guidewire into the intended vein and obtained within three punctures); (8) guidewire advancing difficulties; (9) venous collapse rate (defined as the proportion of patients in whom the vein was collapsed; a vein was said to be collapsed if the visually estimated diameter varies by more than 50% with respiratory movements); (10). Adverse events were evaluated by the rates of arterial puncture, hematoma, pneumothorax and catheter misplacements (Additional file 2). An investigator independently assessed the primary and secondary outcomes.

### Statistical analysis

Statistical analyses were performed using SPSS 21.0 software (SPSS, Inc., Chicago, IL, USA).

Sample size was calculated assuming a proportion of first attempt success rate not less than 0.75 per group. With an alpha value of 0.05 and a power of 85%, inclusion of 114 patients in each group were required to detect a difference not less than 0.15 in the proportion of first attempt success rate between groups (two-tailed test). Considering a dropout rate of 10%, we enrolled 125 patients in each group.

It was estimated that 20 observations were required to assess intra- and inter-observer reliability using three observers (three residents). Intra- and inter-observer reliability were checked by computing intraclass correlation coefficient with 95% confidence interval (CI). There was high inter and intra observer reliability, with no evidence of observer bias in the analysis of all measurements.

Data were presented using count number, percentages, means and standard deviations (SD). The Kolmogorov–Smirnov test was applied to test the normality of data. Data between the groups were compared using Chi-square test, Fischer’s exact test or Student’s t test, depending on the nature of the variables. Odds ratio (OR) with 95% CI were calculated accordingly. A two-tailed p value less than 0.05 was considered the threshold for statistical significance.

## Results

**Fig. 3:**
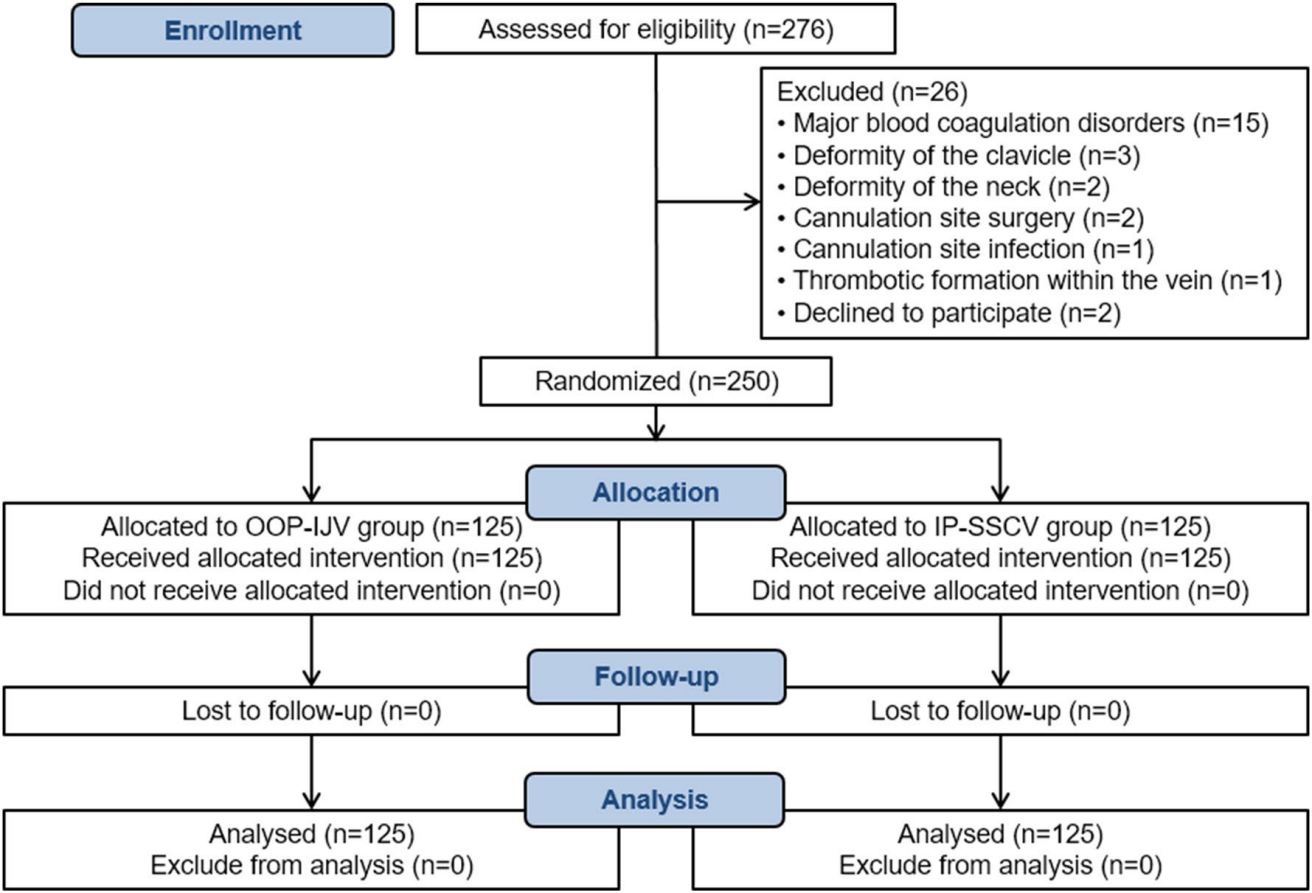
CONSORT flow diagram of the study. *OOP-IJV* out-of-plane internal jugular vein*, IP-SSCV* in-plane supraclavicular subclavian vein

Two hundred and fifty procedures were analyzed. Each group had a total of 125 procedures (Fig. 3). There were no significant differences in patient characteristics and clinical data between the two groups as shown in Table 1. We performed 93 right-side (74.4%) in OOP-IJV group and 74 right-side (59.2%) in IP-SSCV group catheterization attempts. We did not have any missing data. The first attempt success rate was significantly higher in the IP-SSCV group (83.2%) compared to the OOP-IJV group (63.2%) (*p* = 0.001). The IP-SCCV group was associated with a longer mean US scanning time (16.54 ± 13.51 vs. 5.26 ± 4.05 s; *p* < 0.001) and venous puncture time (22.41 ± 18.68 vs. 19.55 ± 15.71 s; *p* = 0.19) as well as a shorter insertion time (43.98 ± 26.77 vs. 53.12 ± 40.21 s; *p* = 0.038) than in the OOP-IJV group. We recorded in the IP-SCCV group a fewer number of puncture attempts (1.16 ± 0.39 vs. 1.47 ± 0.71; *p* < 0.001), needle redirections (0.69 ± 0.58 vs. 1.17 ± 0.95; *p* < 0.001) and difficulties in guidewire advancement (2.4% vs. 27.4%; *p* < 0.001) (Additional file 3). No significant difference was observed between the two groups regarding adverse events. Hematoma at puncture site was the most frequent early complication with a significant difference between the OOP-IJV (11.2%) and IP-SCCV (4%) groups (*p* = 0.03). In the IP-SSCV group, only one pneumothorax occurred requiring chest tube insertion. Misplacement of the CVC was observed in one patient in the IP-SCCV group. The CVC tip was placed into the contralateral SCV (Table 2).

**Table 1:** Baseline characteristics of the study groups

	OOP-IJV group (*n* = 125)	IP-SSCV group (*n* = 125)	*p*
Age, mean ± SD, years	51.99 ± 18.27	49.77 ± 19.18	0.34
Gender ratio, male/female	1.5	2.3	0.11
Body mass index, mean ± SD, kg/m^2^	26.84 ± 5.36	25.93 ± 6.28	0.21
Comorbidities, *n* (%)			
Hypertension	35 (28)	38 (30.4)	0.67
Diabetes mellitus	24 (19.2)	22 (17.6)	0.74
Ischemic heart disease	16 (12.8)	18 (14.4)	0.71
COPD/Asthma	7 (5.6)	8 (6.4)	0.79
Chronic kidney disease	3 (2.4)	2 (1.6)	0.65
Admission type, *n* (%)			
Trauma	46 (36.8)	49 (39.2)	0.69
Medical	42 (33.6)	43 (34.4)	0.89
Postoperative	37 (29.6)	33 (26.4)	0.57
Presence of risk factors for difficult venous cannulation, *n* (%)	17 (13.6)	20 (16)	0.59
Mechanical ventilation during line placement, *n* (%)	87 (69.4)	83 (66.4)	0.62
SOFA score at randomization, mean ± SD	7.91 ± 2.38	7.69 ± 2.83	0.5

*SD* Standard deviation, *COPD* chronic obstructive pulmonary disease, *SOFA* sequential organ failure assessment

**Table 2:** Venous cannulation characteristics

	OOP-IJV group (*n* = 125)	IP-SSCV group (*n* = 125)	*p*
Primary outcome			
First attempt success rate (%)	63.2	83.2	0.001
Secondary outcomes			
US scanning time (s)	5.26 ± 4.05	16.54 ± 13.51	< 0.001
Venous puncture time (s)	19.55 ± 15.71	22.41 ± 18.68	0.19
Insertion time (s)	53.12 ± 40.21	43.98 ± 26.77	0.038
Overall access time (s)	57.95 ± 40.78	59.68 ± 36.13	0.73
Mean number of puncture attempts	1.47 ± 0.71	1.16 ± 0.39	< 0.001
Mean number of needle redirections	1.17 ± 0.95	0.69 ± 0.58	< 0.001
Success rate (%)	96.8	98.4	0.68
Guidewire advancing difficulties (*n* (%))	34 (27.4)	3 (2.4)	< 0.001
Venous collapse (*n* (%))	23 (18.4)	3 (2.4)	< 0.001
Adverse events (*n* (%))	17 (13.6)	11 (8.8)	0.22
Pneumothorax	0	1 (0.8)	0.31
Hemothorax	0	0	–
Arterial puncture	3 (2.4)	4 (3.2)	0.7
Hematoma	14 (11.2)	5 (4)	0.03
Catheter malposition	0	1 (0.8)	0.31

The data were reported as mean ± SD or number (%)

The difference is significant at *p* < 0.05

The venous collapse was more frequent in OOP-IJV group than in IP-SSCV group (*p* < 0.001; OR = 9.17, 95%CI [2.68–31.42]). In OOP-IJV group, the venous collapse was associated with higher risk of catheterization failure on first attempt (*p* < 0.001; OR = 28.88, 95%CI [6.25–133.49]) and with higher risk of difficulties in guidewire insertion (*p* < 0.001; OR = 24.19, 95%CI [7.23–80.93]). These associations were not observed in IP-SSCV group.

## Discussion

In this prospective randomized clinical study, the data showed that the IP-SSCV cannulation group had a significantly higher first attempt success rate in addition to a shorter insertion time, a lower puncture attempts and needle redirections and a lower rate of difficulties in guidewire advancement, venous collapse and hematoma at puncture site compared to OOP-IJV cannulation group.

US-guided IJV cannulation can be performed using two techniques: short-axis or long-axis. The short-axis is technically easier than the long-axis view and is the preferred approach for teaching US-guided IJV cannulation [16, 17]. This imaging technique allows simultaneous cross sectional visualization of the IJV, the CCA and surrounding tissues to avoid these structures [18].

In ICU, the SCV is a good alternative to the IJV especially in hypovolemic or unstable patients. There was insufficient evidence to support the use of US-guidance for SCV cannulation via the classic IC approach which requires high levels of training [19]. Yet, supraclavicular approach offers a better long-axis sonographic visualization of the SCV [10]. Most clinical studies on US-guided IP-SSCV access have been conducted in the pediatric population particularly for BCV cannulation [20, 21]. Data in adults are lacking.

In our study, the US-guided catheterizations were performed by inexperienced residents in training. That is why we have chosen to compare the routinely used short-axis OOP-IJV with the long-axis IP-SSV cannulation. We found that the first attempt success rate was significantly higher in IP-SSCV group compared to OOP-IJV group (83.2% vs. 63.2%; *p* = 0.001). These results are consistent with those reported in the prospective study conducted by Oulego-Erroz et al. comparing the IP-BCV and the OOP-IJV cannulation in critically ill children and reporting a first attempt success rate of 73% and 37.5%, respectively (*p* = 0.017) [22]. In another retrospective cohort involving elective central venous cannulation, Beccaria et al. mentioned a higher first attempt success rate in the BCV group (90%) than in the IJV group (85%) [23]. In fact, the SCV has several anatomical advantages. Due to its large diameter, its intrathoracic position and its firm attachment to adjacent bony structures, the SCV remains patent and stable regardless of the hemodynamic and respiratory status which facilitates the venous access. The supraclavicular approach enables obtaining a good sonographic visualization of the SCV. It also offers the advantage of an easier maintaining of the long-axis view of the SCV given the anatomical features of the supraclavicular fossa allowing stabilization of the US probe against the clavicle [10, 24].

In contrast, the IJV may be difficult to cannulate since it is a superficial vein that tends to collapse under probe or needle pressure or with respiratory movements, particularly in critically ill patients with severe hypovolemia [25]. These physiological features have been well demonstrated in our study, since the venous collapse was significantly more frequent in OOP-IJV group compared to IP-SSCV group. In addition, the venous collapse was associated with a higher risk of first attempt failure of catheterization in the OOP-IJV group. This association was not observed in the IP-SSCV group.

The number of puncture attempts was significantly higher in the OOP-IJV group than the IP-SSCV group. This finding could be explained by the collapsibility of the IJV and by the difficulties experienced during guidewire insertion in the OOP-IJV group, leading to multiple punctures. Indeed, the OOP-IJV group was associated with a significantly higher rate of difficulties in guidewire insertion, compared to IP-SSCV group. We believe that the difficulties encountered in the OOP-IJV group could be explained by the collapsibility of the IJV and the US-guidance used imaging technique. In a prospective randomized study, Batllori et al. reported that the short-axis OOP approach for IJV cannulation was frequently associated with posterior wall puncture of the vein and the passage of the guidewire in the extravascular tissues, resulting in difficulties in guidewire advancement [6]. In fact, the needle is viewed in cross section during the OOP approach. The echogenic point in the center of the vein may not necessarily be the needle tip [3]. The real needle tip could be deeper. Therefore, the operator may inadvertently pass through both vein’s walls. In contrast, the IP approach allows direct real-time sonographic visualization of the whole course of the needle. Furthermore, the supraclavicular SCV puncture is close to the BCV (and thus to the superior vena cava) which is larger than IJV leading to fewer difficulties in guidewire advancement in the IP-SSCV group.

Although the mean insertion time was significantly shorter in the IP-SSCV group, our study showed a comparable overall access time between the two groups. This result could be explained by the longer scanning time observed in the IP-SSCV group compared to OOP-IJV group since the US scanning in the IP-SSCV group began by obtaining the short-axis view of the IJV before translating the US probe towards the supraclavicular fossa, as described in previous studies [26].

Our study revealed a higher incidence of adverse events in the OOP-IJV group compared to IP-SSCV group, without significant difference. However, we observed a significantly higher incidence of hematoma at puncture site in the OOP-IJV group, linked to the significantly higher number of puncture attempts in the same group, as reported by Björkander et al. [27]. In literature, the SCV route was associated with a higher incidence of pneumothorax and hemothorax [28]. In our study, we recorded only one pneumothorax in the IP-SSCV group and no hemothoraces occurred. In fact, in the SCV catheterization, the needle trajectory is parallel to the pleura [29] and the IP technique allows not only the detection of the needle tip advancement within the vein, but also a good visualization of the pleura, decreasing by that the risk of pleural puncture [30].

Our study had some limitations. First, cannulations were performed by three residents in training, inexperienced in US-guided CVC placement. Furthermore, we have not measured the veins’ diameters through the respiratory cycle and the evaluation of the venous collapse was then subjective. In addition, it could be interesting if we studied long-term complications (thrombotic and infectious complications).

## Conclusions

In adult ICU, the IP-SSCV cannulation is a safe and effective technique to performing and teaching CVC insertion with a higher first attempt success rate, a lower rate of difficulties in guidewire insertion and incidence of hematoma at puncture site than with the OOP-IJV cannulation, even if performed by inexperienced operators. The IP-SSCV approach provides a useful alternative technique for central venous catheterization. Further clinical studies are needed to recommend its routine use in daily practice.

## Supplementary Information

**Additional file 1: **Movie file of ultrasound subclavian vein catheterization using supraclavicular approach in adult patient.

**Additional file 2: ** Study protocol.

**Additional file 3: **Main results: comparison between groups.

## Data Availability

The datasets used during the current study are available from the corresponding author on reasonable request.

## Abbreviations

CVC: Central venous catheter
IJV: Internal jugular vein
SCV: Subclavian vein
US: Ultrasound
OOP: Out-of-plane
IC: Infraclavicular
IP: In-plane
ICU: Intensive care unit
CCA: Common carotid artery
BCV: Brachiocephalic vein
SD: Standard deviations
OR: Odds ratio
CI: Confidence interval
