# Supplementary figures and images for: Comparison of ultrasound-guided internal jugular vein and supraclavicular subclavian vein catheterization in critically ill patients: a prospective, randomized clinical trial
Source: Ann Intensive Care. 2022 Oct 1;12:91. doi: 10.1186/s13613-022-01065-x (PMC9526766; doi:10.1186/s13613-022-01065-x)

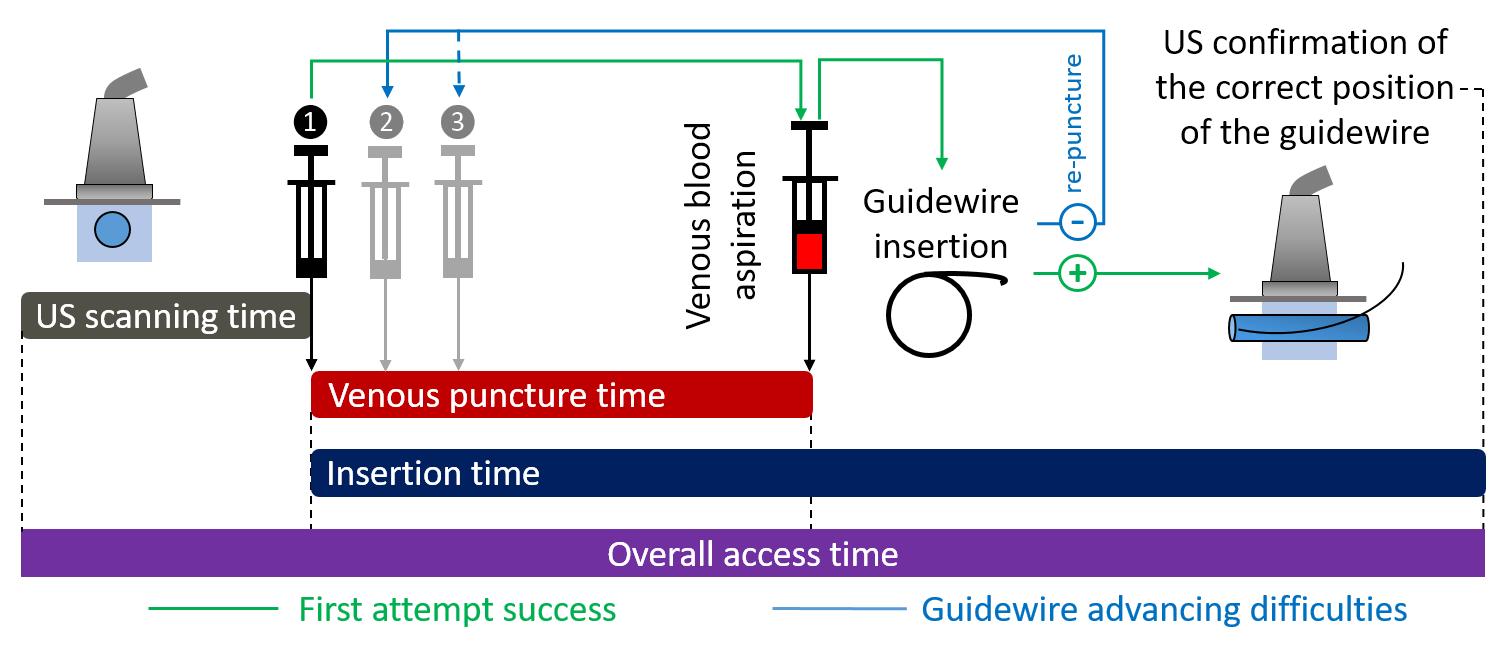

Supplement: Supplementary file 2 — Additional file 2: Study protocol. [file 13613_2022_1065_MOESM2_ESM.png]

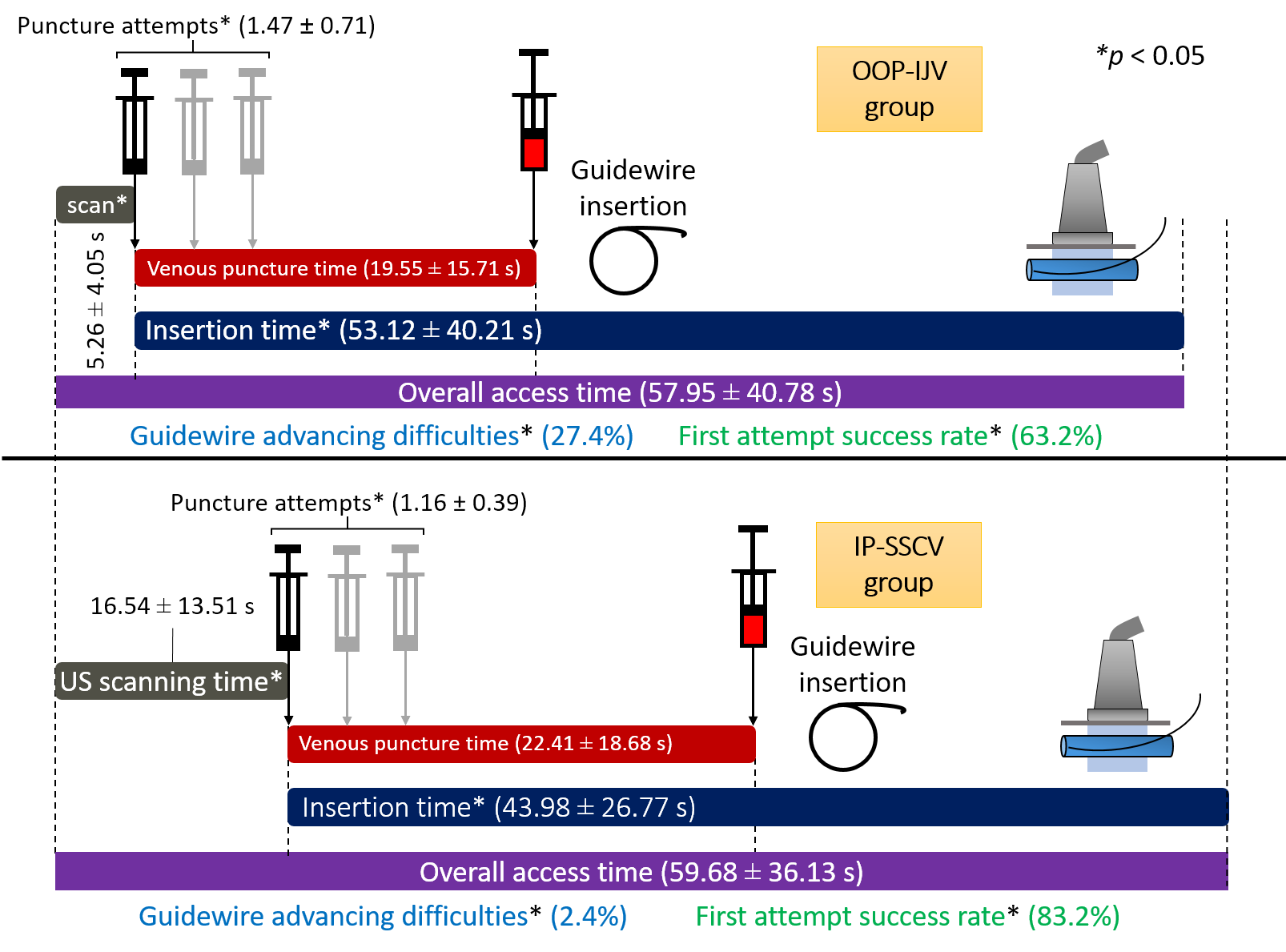

Supplement: Supplementary file 3 — Additional file 3: Main results: comparison between groups. [file 13613_2022_1065_MOESM3_ESM.png]
